# Medikamenteninduzierte Hyperreaktivitätserkrankungen: Pulmonale Manifestationen und radiologische Charakteristika

**DOI:** 10.1007/s00117-026-01565-y

**Published:** 2026-02-16

**Authors:** Justus E. Roos, Lukas Ebner

**Affiliations:** 1 Institut für Radiologie und Nuklearmedizin, Luzerner Kantonsspital, Universitäres Lehr- und Forschungsspital, Universität Luzern, Luzern, Schweiz; 2 Institut für diagnostische, interventionelle und pädiatrische Radiologie, Inselspital Bern, Universtitätsspital Bern, University of Bern, Bern, Schweiz; 3 Radiologie , Beau-Site, Hirslanden, Bern, Schweiz

**Keywords:** Hypersensitivitätspneumonitis, Drug reaction with eosinophilia and systemic symptoms, Eosinophile Pneumonie, Organisierende Pneumonie, Hochauflösende Computertomographie, Hypersensitivity pneumonitis, Drug reaction with eosinophilia and systemic symptoms, Eosinophilic pneumonia, Organising pneumonia, High-resolution computed tomography

## Abstract

Medikamenteninduzierte Überempfindlichkeitsreaktionen (MIHR) stellen ein breites Spektrum immunvermittelter Erkrankungen dar, die nach Exposition gegenüber Arzneimitteln auftreten und verschiedene Organsysteme betreffen können. Zu den wichtigsten Entitäten zählen das DRESS-Syndrom („drug reaction with eosinophilia and systemic symptoms“), das Stevens-Johnson-Syndrom (SJS) und die toxische epidermale Nekrolyse (TEN, Lyell-Syndrom), die Serumkrankheit, medikamenteninduzierte eosinophile Pneumonien sowie Hypersensitivitätspneumonitiden. Pulmonale Manifestationen sind dabei häufig und können den klinischen Verlauf entscheidend beeinflussen. Dieser Übersichtsartikel erläutert die immunologischen Grundlagen, die Pathogenese und Ätiologie der medikamenteninduzierten Hyperreaktivitätserkrankungen, beschreibt ihre klinische Relevanz und stellt die typischen thorakalen Manifestationen und Bildmuster in der Computertomographie (CT) des Thorax dar. Im Vordergrund steht die Bedeutung der radiologischen Diagnostik bei der frühzeitigen Erkennung und Differenzierung dieser potenziell lebensbedrohlichen Krankheitsbilder.

## Abstract (en)

Drug-induced hypersensitivity reactions (DIHR) represent a broad spectrum of immune-mediated disorders that occur after exposure to medications and can affect various organ systems. The most important entities include drug reaction with eosinophilia and systemic symptoms (DRESS) syndrome, Stevens–Johnson syndrome (SJS) and toxic epidermal necrolysis (TEN, Lyell’s syndrome), serum sickness, drug-induced eosinophilic pneumonias, and hypersensitivity pneumonitis. Pulmonary manifestations are common and can have a decisive impact on the clinical course. This review article explains the immunological foundations, pathogenesis, and etiology of drug-induced hyperreactivity disorders, describes their clinical relevance, and presents the typical thoracic manifestations and imaging patterns in chest computed tomography (CT). The focus is on the importance of radiological diagnostics for the early detection and differentiation of these potentially life-threatening conditions.

Arzneimittelbedingte Überempfindlichkeitsreaktionen sind seltene, aber klinisch relevante Ursachen akuter und subakuter interstitieller oder alveolärer Lungenerkrankungen. Während die meisten Nebenwirkungen von Medikamenten pharmakologisch erklärbar und dosisabhängig sind, handelt es sich bei den hier beschriebenen Entitäten um immunologisch vermittelte, idiosynkratische Reaktionen, die nach wiederholter oder sogar erstmaliger Exposition auftreten können.

Prinzipiell lassen sich medikamentenbedingte Reaktionen schematisch in 3 Hauptgruppen unterteilen, wie in Abb. 1 dargestellt. Erstens existieren pharmakologisch getriggerte Reaktionen, die direkt auf die Wirkmechanismen des Arzneistoffs zurückzuführen sind. Zweitens finden sich immunologisch vermittelte Reaktionen, bei denen eine spezifische Aktivierung des adaptiven Immunsystems eine zentrale Rolle spielt. Drittens werden pseudoallergische Reaktionen abgegrenzt, die zwar entzündliche Zellkomponenten und ähnliche klinische Manifestationen aufweisen, jedoch ohne primäre Beteiligung immunologischer Effektormechanismen entstehen.

**Abb. 1:**
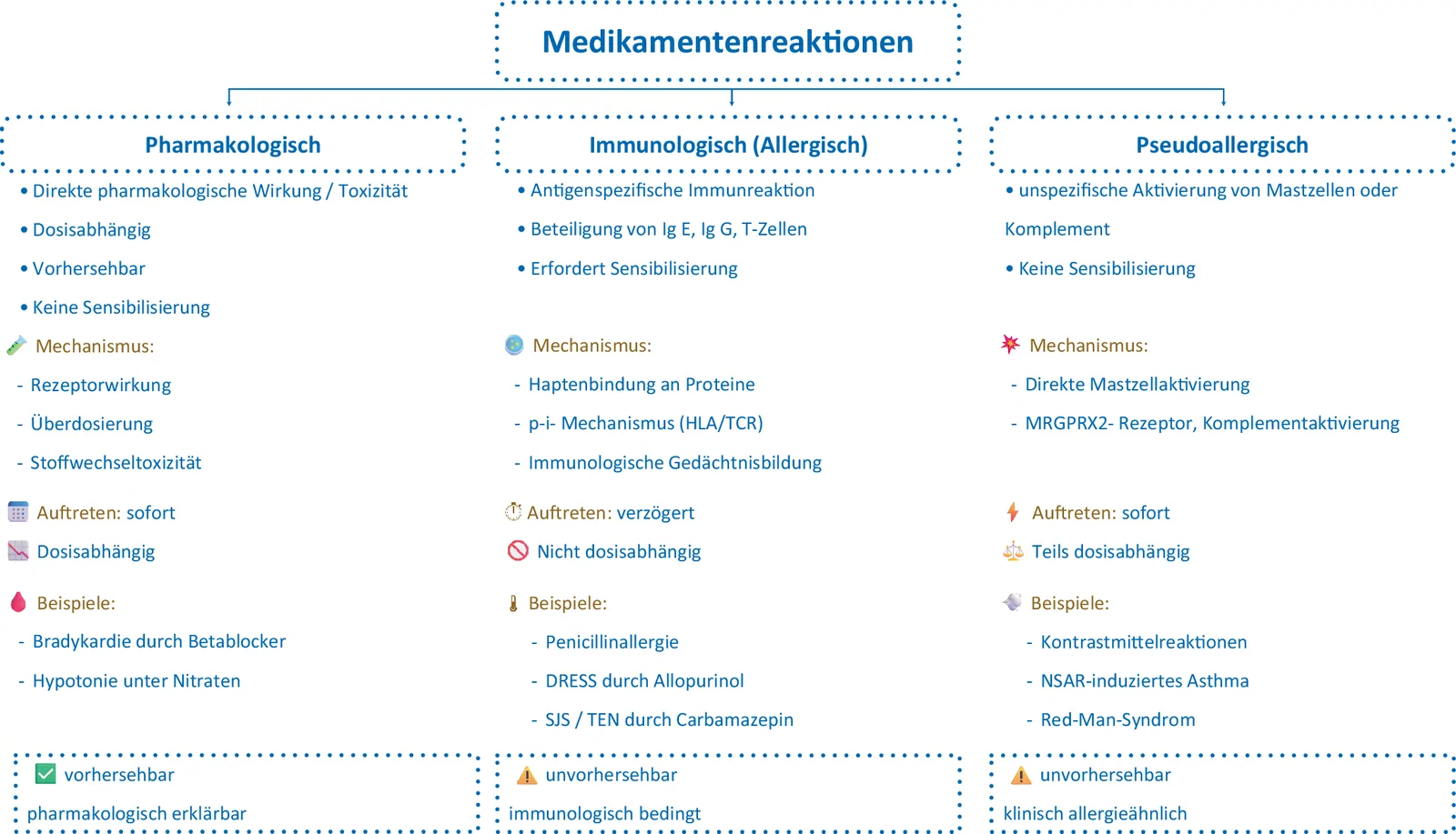
*p‑i-Mechanismus* („pharmacological interaction with immune receptors“). Das Medikament bindet direkt, nichtkovalent und reversibel an den T‑Zell-Rezeptor (*TCR*) oder an das *HLA*-Molekül (humanes Leukozytenantigen) auf der antigenpräsentierenden Zelle. Dadurch wird der TCR pharmakologisch stimuliert, ohne dass das Medikament zuvor an ein körpereigenes Protein gebunden oder als klassisches Antigen prozessiert werden muss. Diese direkte Interaktion führt zur Aktivierung von T‑Zellen und damit zu einer immunologischen Reaktion. *MRGPRX2-Rezeptor* (Mas-related G protein-coupled receptor X2) liegt auf den Mastzellen und kann direkt durch bestimmte Medikamente (auch Kontrastmittel) aktiviert werden – ohne Beteiligung von IgE-Antikörpern oder anderer klassischer immunologischer Mechanismen. *DRESS* Drug reaction with eosinophilia and systemic symptoms, *IgE* Immunglobulin E, *NSAR* nichtsteroidale Antirheumatika, *SJS* Stevens-Johnson-Syndrom, *TEN* toxische epidermale Nekrolyse

Arzneimittelüberempfindlichkeit ist ein Begriff, der über die klassische Arzneimittelallergie hinausgeht. Immunreaktionen und entsprechende klinische Symptome können auch auftreten, wenn Medikamente direkt an Immunrezeptoren wie HLA(humane Leukozytenantigene)- oder T‑Zell-Rezeptoren binden oder wenn Entzündungszellen durch Medikamenten-Rezeptor- oder Medikamenten-Enzym-Interaktionen stimuliert werden (sog. Pseudoallergie). Ein erheblicher Teil der arzneimittelbedingten Überempfindlichkeitsreaktionen folgt den pharmakologischen anstatt immunologischen Mechanismen und zeigt Merkmale einer Typ-A-Reaktion (vorhersehbare, dosisabhängige Reaktion). Dies beeinflusst den klinischen Verlauf, das Management, die Vorhersagbarkeit, den Dosis-Effekt-Zusammenhang und die Kreuzreaktivität solcher Reaktionen und sollte daher bei der klinischen Beurteilung berücksichtigt werden [1].

Das „drug reaction with eosinophilia and systemic symptoms“ (DRESS), oder auch „drug-induced hypersensitivity syndrome“ (DiHS) genannt, ist eine schwere, seltene und T‑Zell-vermittelte Überempfindlichkeitsreaktion. Sie äußert sich in unterschiedlichen Kombinationen von Hautausschlag, Fieber, Gesichtsschwellung, Lymphknotenschwellung, Veränderungen im Blutbild (z. B. Eosinophilie) sowie Beteiligung innerer Organe.

Im Vergleich zu den anderen schweren arzneimittelbedingten Hautreaktionen (SCARs) – wie dem Stevens-Johnson-Syndrom (SJS), der toxischen epidermalen Nekrolyse (TEN) oder der akuten generalisierten exanthematischen Pustulose (AGEP) – zeigt DRESS ein sehr variables klinisches Erscheinungsbild [2].

Das klinische Spektrum reicht von milden makulopapulösen Exanthemen bis zu schweren systemischen Erkrankungen mit Multiorganversagen. Das DRESS-Syndrom gilt als prototypische Form einer immunologischen, durch T‑Zellen getriggerten medikamenteninduzierten Hyperreaktivität und ist durch eine ausgeprägte Eosinophilie, Lymphadenopathie und eine oft verzögerte, systemische Organbeteiligung gekennzeichnet [3]. Ähnliche Mechanismen spielen auch bei anderen Entitäten wie dem Stevens-Johnson-Syndrom (SJS), der Serumkrankheit oder der medikamenteninduzierten eosinophilen Pneumonie eine Rolle [4].

Auch wenn initiale Manifestationen fast immer kutan auftreten, sind Organmanifestationen eine schwerwiegende Komplikation. Vor allem betroffen sind u. a. die Leber (> 50 %) und Nieren; grundsätzlich kann aber jedes Organsystem betroffen sein [5]. Je nach Pathomechanismus können alveoläre, interstitielle oder vaskulitische Muster auftreten. Radiologinnen und Radiologen kommt hier eine Schlüsselrolle zu: Sie sind oft die ersten, die die inflammatorischen Veränderungen im Thorax erkennen und so eine differenzierte Diagnose zwischen infektiösen, immunlogischen und medikamentösen Ursachen ermöglichen.

## Ätiologie

Eine Vielzahl von Medikamenten ist in der Lage, eine Hyperreaktivität zu induzieren. Besonders häufig betroffen sind antikonvulsive Substanzen wie Carbamazepin, Phenytoin oder Lamotrigin, Antibiotika – insbesondere Sulfonamide, Minocyclin und Vancomycin – sowie Allopurinol, das in europäischen Kohorten eine der häufigsten Auslöser des DRESS-Syndroms darstellt [3, 6]. Darüber hinaus wurden antiretrovirale Medikamente wie Abacavir und Nevirapin, nichtsteroidale Antiphlogistika, Zytostatika (z. B. Methotrexat, Bleomycin) sowie moderne Immuncheckpoint-Inhibitoren (ICI) als Auslöser beschrieben (Tab. 1).

**Tab. 1:** Arzneimittelüberempfindlichkeitsreaktionen – Übersicht. (Mod. nach [1])

Unterscheidungsmerkmal	Beschreibung/Beispiele
Klinische Symptome – zeitlicher Verlauf nach Medikamenteneinnahme	< 1 bis < 6 h:
	– Urtikaria (Nesselsucht)
	– Angioödem
	– Anaphylaxie (meist < 1 h)
	→ vermittelt durch IgE oder Pseudoallergie
	> 6 h, oft Tage:
	– Vielfältige Symptome durch T‑Zellen oder IgG vermittelt
Immunologische Mechanismen (nach Coombs und Gell)	Typ I (IgE-vermittelt):
	– Schnell, meist < 1 h
	– Urtikaria, Anaphylaxie
	Typ II (IgG, zytotoxisch):
	– Verzögert, nach 1–14 Tagen
	– Blutbildveränderungen (Zytopenien)
	Typ III (IgG, Immunkomplex):
	– Verzögert, nach 2–14 Tagen
	– Vaskulitis, Serumkrankheit
	Typ IV (T-Zell-vermittelt):
	– Verzögert, nach 2 bis > 20 Tagen
	– Unterschiedliche Exantheme, Hepatitis
Typische auslösende Medikamente	NSAR:
	– Atemwegs- und/oder Hautreaktionen, Exazerbationen
	– Meist Pseudoallergie
	Antikonvulsiva:
	– DRESS, SJS/TEN
	– Meist durch p‑i-Mechanismus, häufig assoziiert mit bestimmten HLA-Allelen
	Biologika (Proteinpräparate):
	– Infusionsreaktionen, vermittelt durch IgE, IgG, Komplement oder Neutralisation (Verlust der Wirksamkeit)
Wirkungsweise des Medikaments auf Immun‑/Entzündungszellen	Allergisch (IgE-vermittelt):
	– Penicillinallergie, Kontaktdermatitis
	– Kombinierte IgE- und T‑Zell-Reaktionen
	p‑i-Mechanismus (HLA, TCR):
	– Nur T‑Zell-Reaktionen
	Pseudoallergie (z. B. über MRGPRX auf Mastzellen oder Enzymblockade):
	– Urtikaria, Anaphylaxie, Bronchospasmus (bei zugrunde liegender Entzündung)
	– Keine spezifischen IgE- oder T‑Zell-Nachweise

*DRESS* Drug reaction with eosinophilia and systemic symptoms,* HLA* humane Leukozytenantigene,* IgE* Immunglobulin E, *IgG* Immunglobulin G, *MRGPRX* Mas-related G protein-coupled receptor X2, *NSAR* nichtsteroidale Antirheumatika, *p‑i *pharmacological interaction with immune receptors,* SJS* Stevens-Johnson-Syndrom, *TCR* T-Zell-Rezeptor*, TEN* toxische epidermale Nekrolyse

## Immunologische Grundlagen und Pathogenese

### Klinische Relevanz und systemische Manifestationen

Die klinische Präsentation variiert erheblich. Das DRESS-Syndrom manifestiert sich typischerweise mit Fieber, makulopapulösem Exanthem, Lymphadenopathie, Eosinophilie und Organbeteiligung. Neben der Leber (in bis zu 50 % der Fälle) sind Niere, Lunge und Herz häufig betroffen [3]. Die Mortalität liegt bei etwa 5–10 %.

Die Abgrenzung zwischen medikamenteninduzierten Hyperreaktivitätserkrankungen und der „drug-induced interstitial lung disease“ (DILD) ist klinisch und radiologisch von besonderer Bedeutung, da beide Entitäten überlappende, aber unterschiedliche pathogenetische Mechanismen und Bildmuster aufweisen. Unter dem Begriff DILD wird ein breites Spektrum von nichtinfektiösen, medikamentenbedingten pulmonalen Reaktionen zusammengefasst, die strukturell zu einer Lungenbeteiligung führen. Diese umfasst klassische, toxisch-fibrosierende Mechanismen (z. B. durch Amiodaron, Bleomycin oder Methotrexat), aber auch immunologisch vermittelte Formen, bei denen eine Hypersensitivitätskomponente besteht [7].

Im Gegensatz dazu beruhen die medikamenteninduzierten Hyperreaktivitätserkrankungen (MIHR), wie DRESS, SJS/TEN oder Serumkrankheit, definitionsgemäß auf einer adaptiven, antigenspezifischen Immunantwort. Diese Reaktionen sind durch Sensibilisierung und T‑Zell-vermittelte Entzündungsvorgänge charakterisiert, die systemisch auftreten und sich nicht nur auf das Lungenparenchym beschränken [4].

**Abb. 2:**
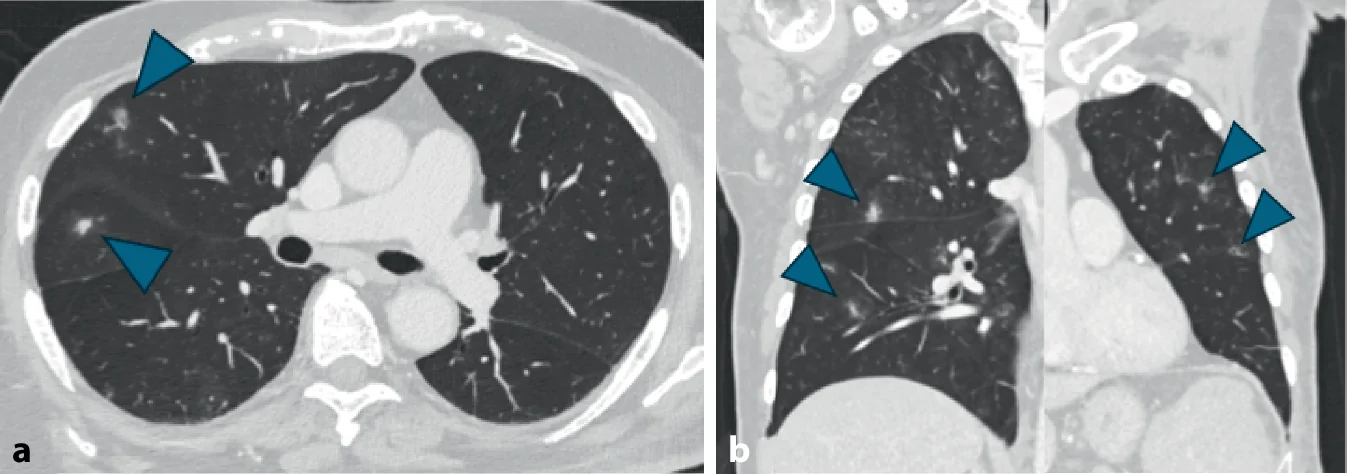
DRESS-Syndrom („drug reaction with eosinophilia and systemic symptoms“) nach Einnahme von Mabthera® (Rituximab) bei Mantelzell-Lymphom. 56-jähriger Patient mit bekanntem Mantelzell-Lymphom, CT-Thorax in axialer (**a**) und koronarer (**b**) Schichtführung. Fünf Wochen nach Gabe von Mabthera Auftreten von Dyspnoe und allgemeinen Symptomen. Klinisch konnte die Diagnose eines DRESS gestellt werden. Radiologisch imponieren diskrete, neu aufgetretene, pulmonale Nodularitäten mit teils diskretem Milchglas-Halo (*Pfeilspitzen* in **a** und **b**). Bei Eosinophilie wurde der Verdacht auf umschriebene Infiltrate im Rahmen des DRESS geäußert. Die Veränderungen waren nach entsprechender Therapie vollständig regredient

In der Praxis überschneiden sich die Entitäten häufig: Das DRESS-Syndrom kann sich radiologisch als interstitielle Pneumonitis präsentieren, und umgekehrt weisen viele Fälle von DILD immunologische Reaktionsmuster der Lungen auf (Abb. 2 und 3). Toxisch bedingte DILD (z. B. durch Bleomycin oder Amiodaron) zeigen histologisch eine diffuse alveoläre Schädigung mit Hyalinmembranbildung und fibroblastischer Organisation. Die radiologischen Korrelate sind bilaterale Milchglasverschattungen, retikuläre Veränderungen und, bei chronischem Verlauf, fibrosierende Umbauten [8]. Im Gegensatz dazu sind immunvermittelte DILD durch lymphozytäre und eosinophile Infiltration gekennzeichnet. Diese entsprechen häufig einer medikamenteninduzierten Hypersensitivitätspneumonitis oder eosinophilen Pneumonie, mit Milchglasarealen, zentrilobulären Noduli oder Muster einer organisierenden Pulmonie (OP).

**Abb. 3:**
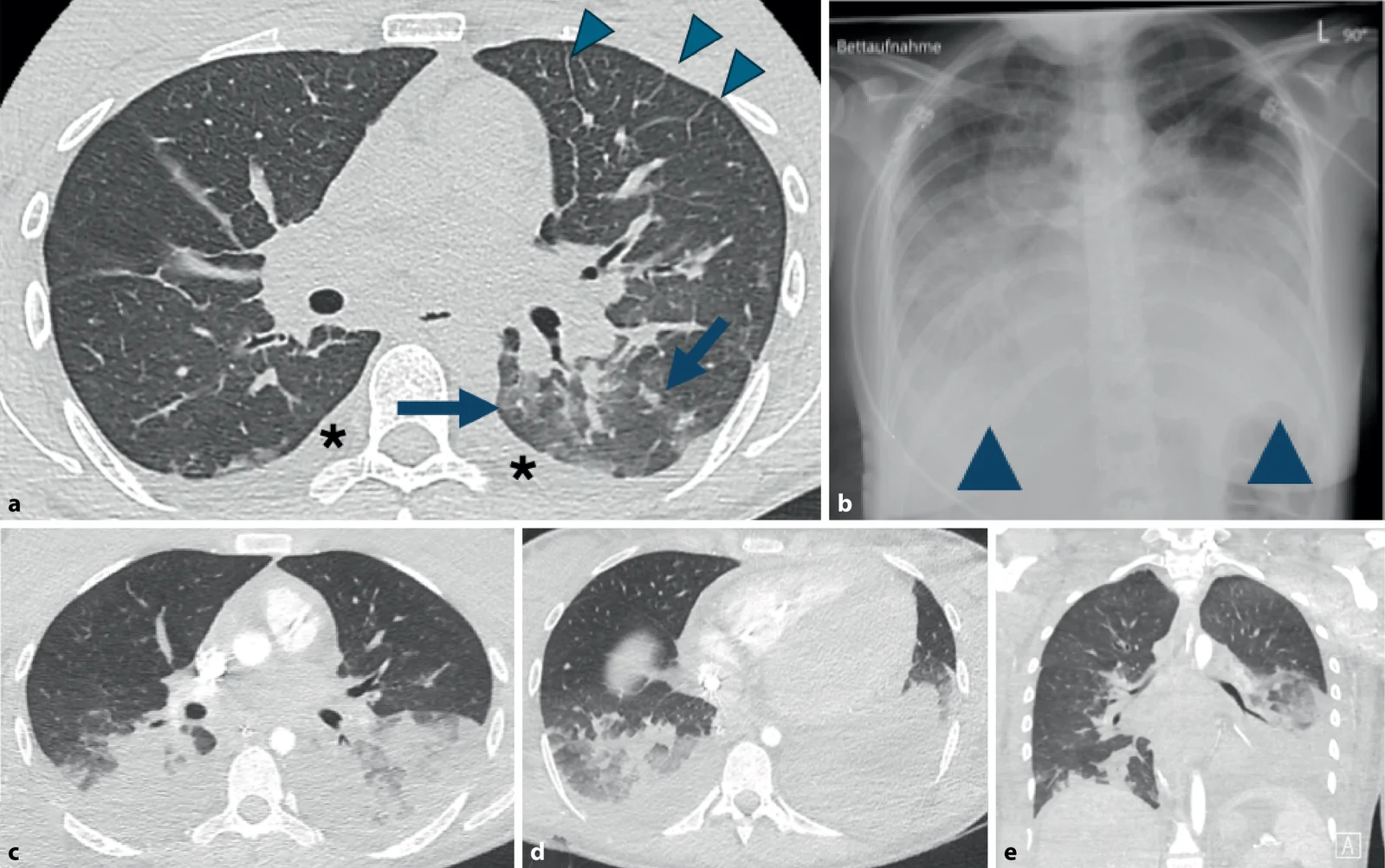
26-jährige Patientin mit DRESS („drug reaction with eosinophilia and systemic symptoms“) und Status nach Reanimation im Spital mit Breitkomplex-Tachykardie. **a** CT-Thorax axial und koronar, bilaterale, Milchglasopazitäten v. a. im linken Unterlappen (*Pfeile*). Darüber hinaus imponieren bilaterale, links betonte Interlobularsepten, Verdickungen (*Pfeilspitzen*) und beidseitige Pleuraergüsse (*Stern*). Zusätzlich bestehen Teilatelektasen in beiden Unterlappen. **b** Röntgen-Thorax im 2‑tägigen Verlauf: deutliche Zunahme der beidseitigen Pleuraergüsse mit positivem Silhouetten-Phänomen zur Herzkontur und Zwerchfell (*Pfeilspitzen*). Formal ist dieses Bild nicht von einem kardialen Ödem z. B. im Rahmen einer Linksherzinsuffizienz zu unterscheiden. **c** Im weiteren Verlauf zeigte sich sowohl klinisch als auch radiologisch eine deutliche Befundverschlechterung (**d**, **e**). Die Patientin entwickelte zusätzlich eine medikamentös-induzierte nekrotisierende Myokarditis. Trotz notfallmäßig durchgeführter Herztransplantation verstarb die Patientin infolge eines zusätzlichen Infekts

### Radiologische Manifestationen

Die radiologische Präsentation medikamenteninduzierter Lungenerkrankungen ist vielgestaltig und reicht von dezenten Milchglasverschattungen bis zu schweren ARDS-ähnlichen Mustern („acute respiratory distress syndrome“). Die hochauflösende Computertomographie (HRCT) gilt als diagnostischer Goldstandard. Sie ermöglicht die Beurteilung des Verteilungsmusters (zentral vs. peripher) der betroffenen Strukturen (interstitiell vs. alveolär) sowie des Verlaufs und erlaubt dadurch eine Zuordnung zu spezifischen pathophysiologischen Prozessen.

## Eosinophile Pneumonie (akut oder chronisch)

**Abb. 4:**
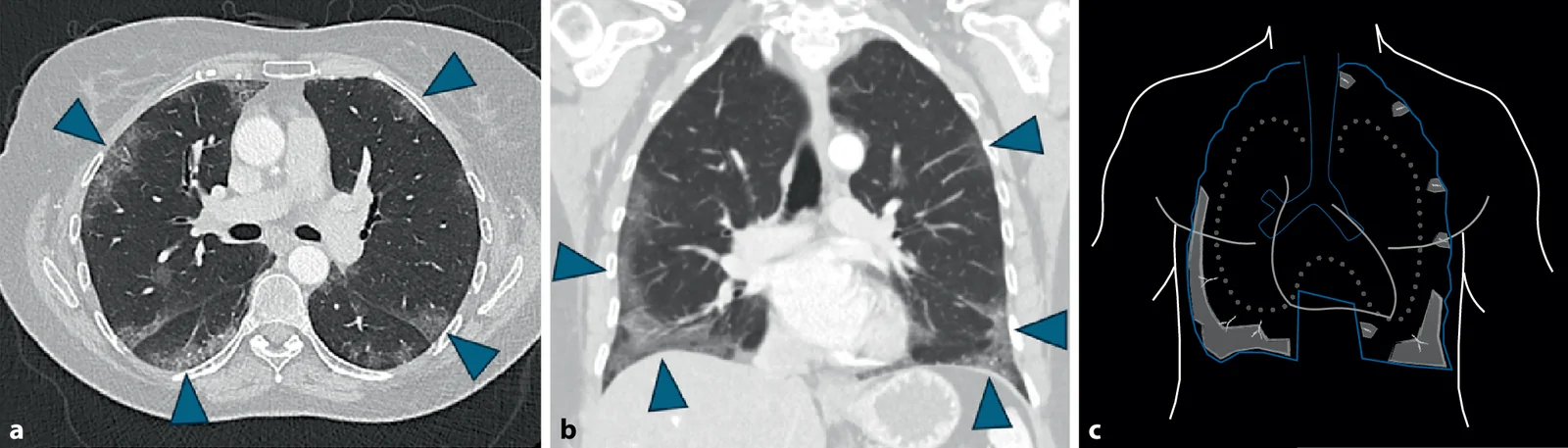
Eosinophile Pneumonie bei Einnahme eines nichtsteroidalen Antirheumatikums (NSAR). 47-jährige Patientin bei schwerwiegenden Periarthropathien unter NSAR-Therapie (Naproxen). Innerhalb von Tagen Auftreten von Fieber, trockenem Husten und progredienter Dyspnoe. Thorax-CT axial (**a**) und koronal (**b**) zeigen peripher und unterlappenbetonte Milchglasopazitäten (siehe Pfeile). Koronales Schema (**c**) visualisiert die als „inverse pulmonary edema“ bekannte inverse – periphere – Verteilung der Opazitäten. Im Blutbild zeigte sich eine erhöhte Blutsenkung (BSR), erhöhtes C‑reaktives Protein (CRP) und eine periphere Eosinophilie. Mit Absetzen der NSAR-Therapie rasche Besserung der klinischen Symptome

Dieses Muster findet sich häufig bei Medikamenten wie Daptomycin, Minocyclin, Nitrofurantoin oder nichtsteroidalen Antirheumatika (NSAR). In der HRCT dominieren ausgedehnte Milchglasverschattungen mit peripherer Betonung (*Photonegativ des Ödems*) und teils subpleuralen Konsolidierungen. Charakteristisch ist eine relative Aussparung der zentralen Lungenanteile. Typische radiologische Befunde zeigen homogene Verdichtungen und/oder Milchglasverschattungen, meist mit peripherer Verteilung (Abb. 4). Die Veränderungen können wandernd (migratorisch) auftreten und gelegentlich ein *Crazy-paving-Muster* (netzartige Zeichnung durch überlagerte Verdichtung und interlobuläre Septenverdickung) zeigen. Häufig finden sich auch bandförmige Verschattungen, die parallel zur Brustwand verlaufen. Diese Veränderungen treten meist mehr als 2 Monate nach Symptombeginn auf. Seltener werden interlobuläre Septenverdickungen, Knötchen oder Massen, mediastinale Lymphknotenvergrößerungen oder Pleuraergüsse (unter 10 %) beobachtet.

## Organisierende-Pneumonie-ähnliches Muster

Bei DRESS-Syndrom, medikamentöser Hypersensitivitätspneumonitis oder nach Checkpoint-Inhibitor-Therapie treten häufig Muster einer organisierenden Pneumonie (OP) auf.

**Abb. 5:**
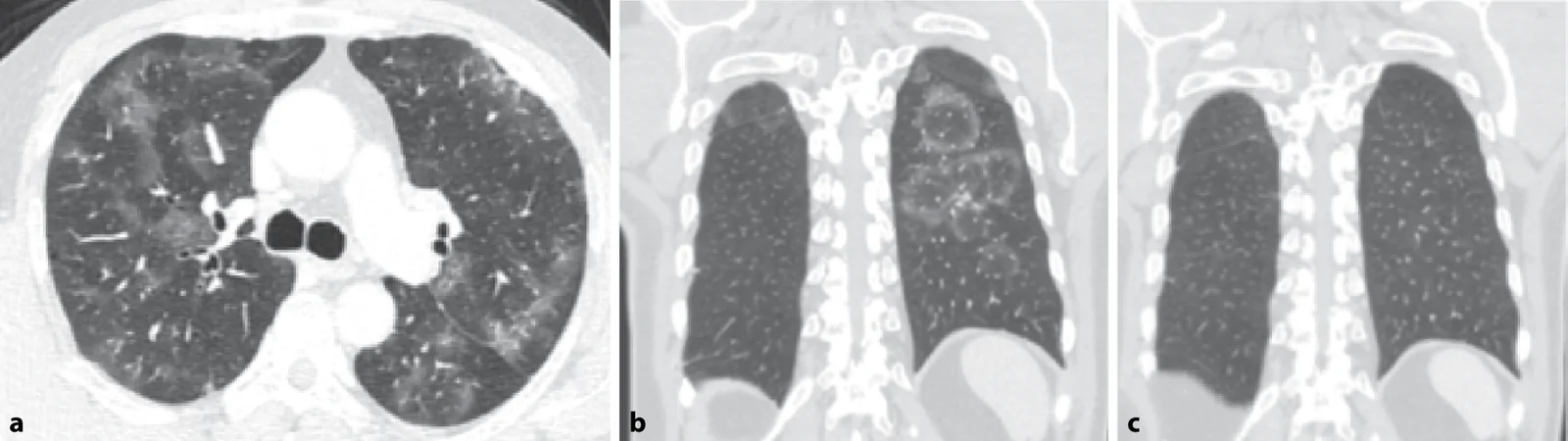
Organisierende Pneumonie (OP) unter Einnahme einer Chemokombinationstherapie mit Carboplatin und Paclitaxel bei einer Patientin mit Ovarialkarzinom; neu aufgetretene Dyspnoe und Husten. In der durchgeführten CT-Thorax zeigen sich bilaterale, arkadenförmige oder perilobuläre Milchglasinfiltrate (**a**, **b**). Die ring- oder arkadenförmigen Infiltrate werden auch als Atoll-Zeichen bezeichnet. Diese sind charakteristisch u. a. für eine OP. Wie in diesem Fall ist die unter Chemotherapie neu aufgetretene Symptomatik sowie das radiologische Bild typisch für eine medikamentös getriggerte OP. Nach Therapiepause sowie Gabe von Steroiden imponieren die perilobulären Milchglasinfiltrate vollständig regredient (**c**)

Die Bildgebung zeigt meist diffuse, beidseitig verstreute periphere oder peribronchiale Konsolidierungen, oft mit unscharfen, bogenförmigen oder polygonalen (perilobulären) Verschattungen um die sekundären Lungenläppchen. Häufig kann ein Reversed-Halo-(Atoll‑)Zeichen beobachtet werden. In einseitigen Fällen kann sich ein einzelner Knoten, eine Raumforderung oder eine Konsolidierung zeigen, gelegentlich auch Milchglasverschattungen. Die Infiltrate können wandernd (migratorisch) auftreten und sprechen häufig gut auf eine Kortikosteroidtherapie an (Abb. 5).

In seltenen Fällen bei Persistenz des schädigenden Agens kann es zu einem Fortschreiten in Richtung einer Lungenfibrose, ähnlich dem Bild einer nichtspezifischen interstitiellen Pneumonie (NSIP), führen. Es wird diskutiert, dass einige NSIP-Fälle möglicherweise eine Folge einer OP darstellen. Dabei können peribronchovaskuläre Milchglasverschattungen, retikuläre Veränderungen und Traktionsbronchiektasen auftreten, gelegentlich mit subpleuraler Aussparung. Kavitationen sind selten.

## Diffuse alveoläre Schädigung (DAD)/ARDS-ähnliches Muster

Dieses Muster findet sich bei schweren Formen des DRESS-Syndroms, beim Stevens-Johnson-Syndrom oder bei toxischen DILD (z. B. Amiodaron, Bleomycin). In der Frühphase zeigen sich typischerweise beidseitige Milchglasverschattungen und/oder Konsolidierungen mit einem anterior-posterioren Verteilungsmuster. Dichtere Verschattungen in nichtabhängigen Lungenabschnitten können auf eine zusätzliche Infektionskomponente hinweisen. Innerhalb der Milchglasareale können bronchiale Dilatationen beobachtet werden, die entweder reversibel sind oder eine Traktionsbronchiektasie widerspiegeln. Pleuraergüsse treten in dieser Phase häufig auf.

In der Spätphase finden sich grobe retikuläre Strukturen und Traktionsbronchiektasien, kombiniert mit diffusen Milchglas- und Netzzeichnungsmustern. In nichtabhängigen Lungenbereichen können transparente Areale (wie Honigwabenmuster oder Bullae) auftreten, die auf eine fibrotische Umwandlung hindeuten. Diese Regionen gelten als anfälliger für Barotraumata als die bereits konsolidierten Lungenabschnitte. Symmetrische Befunde werden häufiger bei einem ARDS infolge extrapulmonaler Ursachen beobachtet.

## Hypersensitivitätspneumonitis-ähnliches Muster

**Abb. 6:**
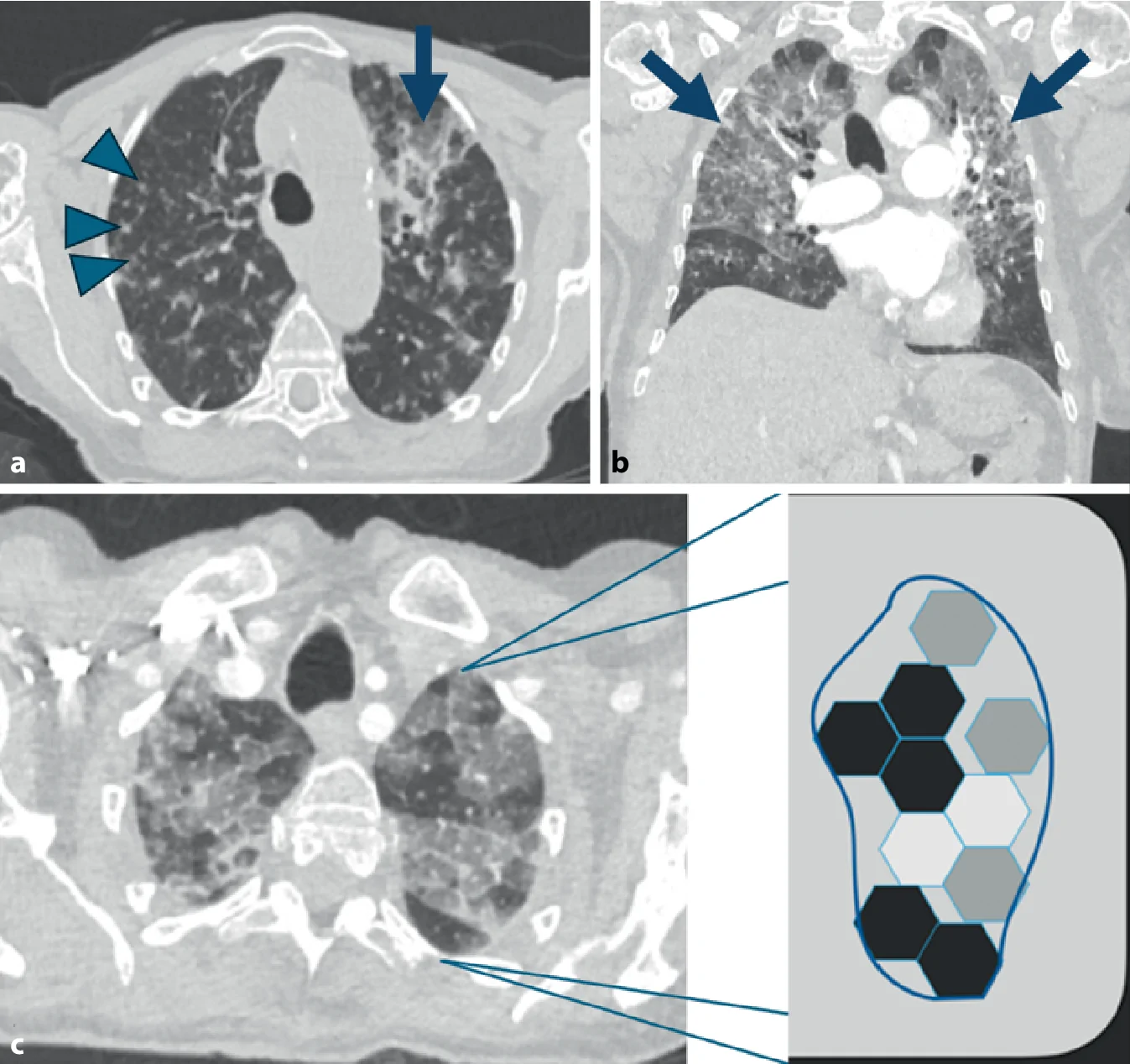
Hypersensitivitätspneumonitis-ähnliches Muster unter Einnahme von Lenalidomid bei multiplem Myelom. 73-jährige Patientin mit vermindertem Allgemeinzustand und progredienter Anstrengungsdyspnoe bei Status nach Stammzelltransplantation und 3‑monatiger Lenalidomid-Therapie bei multiplem Myelom. Initiale antibiotische Breitbandtherapie führte zu keiner Verbesserung der Symptome. Die initiale Thorax-CT-Untersuchung (**a**) – im Rahmen einer Ganzkörper-CT-Untersuchung – zeigte primär oberlappenbetonte zentrilobuläre Nodularität (*Pfeilspitzen*) gemischt mit Michglasverdichtungen, teils konsolidativ (*Pfeile*). In der Anschluss Thorax-CT-5 Tage später (**b**) veränderte sich das Muster in primär flächige Milchglasopazitäten (*Pfeile*) mit einem mosaikartigen Pattern (**c**) von iso-, hypo-, und hyperdensen Veränderungen („three density sign“). Die Oberlappenverteilung blieb bestehen. In der Zytologie der bronchoalveolären Lavage (BAL) fand sich eine ausgeprägte Eosinophilie. Bei fehlendem Erregernachweis wurde eine hoch dosierte Steroidtherapie initiiert, und es zeigte sich eine rasche Regredienz der pulmonalen Infiltrate und der Klinik

Einige Medikamente, darunter Methotrexat, Bortezomib, Bleomycin und Hydroxurea, können eine medikamentöse Hypersensitivitätspneumonitis (HP) auslösen. Die HRCT zeigt zentrilobuläre, milchglasdichte Noduli, teils mit Mosaikperfusion und Air-Trapping. Letzteres ist für die Hypersensitivitätspneumonitis ein typisches Muster und wird als „three density sign“ beschrieben. Hierbei zeigt sich eine mosaikartige Verteilung von normal belüfteten isodensen, hypodensen (durch Air-Trapping bedingten) und hyperdensen (inflammatorisch oder fibrotisch infiltrierten) Arealen ([9, 10]; Abb. 6). Histologisch findet sich eine lymphozytäre Alveolitis mit Bildung nichtverkäsender Granulome. Im Gegensatz zu toxischen DILD sind diese Veränderungen bei frühzeitigem Absetzen des Medikaments meist reversibel.

## Interstitielle und fibrosierende Muster

Vor allem bei toxischen DILD (z. B. Amiodaron, Bleomycin, Methotrexat) dominieren Zeichen einer Fibrose: retikuläre Verdichtungen, Honigwabenstrukturen und Bronchiektasien als Zeichen chronisch-fibrosierender Veränderungen [11]. Die Veränderungen sind oft basal betont und können in interstitielle Pneumonien übergehen. Amiodaron-induzierte Veränderungen zeigen zusätzlich eine erhöhte Lungenattenuation in der HRCT („high-density sign“) aufgrund der Jodakkumulation. Dieses Muster hilft bei der Differenzierung von anderen fibrosierenden ILD.

## Differenzialdiagnose

Wesentliche Differenzialdiagnosen umfassen infektiöse Pneumonien, autoimmune Vaskulitiden, Immuntherapie-assoziierte Pneumonitiden sowie toxische Lungenschäden. Im Gegensatz zu infektiösen Pneumonien fehlen bei medikamenteninduzierten Hyperreaktivitätserkrankungen (MIHR) häufig Erregernachweise und eine rasche klinische Besserung unter Antibiotikatherapie. Autoimmune vaskulitische Erkrankungen wie die eosinophile Granulomatose mit Polyangiitis (EGPA) zeigen typischerweise ANCA-Positivität und extrapulmonale Manifestationen. Eine Immuncheckpoint-Inhibitor-assoziierte Pneumonitis kann bildmorphologisch ähnlich erscheinen, unterscheidet sich jedoch durch den onkologischen Kontext [12].

## Therapie, Verlauf und Nachsorge

Therapeutisch steht das sofortige Absetzen des auslösenden Medikaments im Vordergrund. Systemische Kortikosteroide gelten als Standardbehandlung. In schweren Fällen, insbesondere bei DRESS oder SJS/TEN, kann eine zusätzliche Immunsuppression mit Cyclosporin A oder intravenösen Immunglobulinen erforderlich sein [12, 13].

Radiologisch zeigt sich unter Therapie meist eine rasche Rückbildung der Infiltrate innerhalb von 2 bis 4 Wochen. Eine Verlaufskontrolle mittels HRCT kann nötig sein, um residuelle Veränderungen oder fibrosierende Umbauten zu erfassen.

## Fazit für die Praxis

- Medikamenteninduzierte Hyperreaktivitätserkrankungen (MIHR) sind ein diagnostisch anspruchsvolles, interdisziplinäres Krankheitsbild mit oft charakteristischen pulmonalen Manifestationen.
- Die Kenntnis der immunologischen Grundlagen und der typischen radiologischen Muster ist für Radiologinnen und Radiologen entscheidend, um die Diagnose frühzeitig zu stellen und eine adäquate Therapie einzuleiten.
- Die Einordnung der Bildbefunde im zeitlichen Kontext zur Medikamenteneinnahme, die Abgrenzung zu infektiösen und autoimmunen Prozessen und die enge interdisziplinäre Zusammenarbeit mit Kliniken für Dermatologie, Immunologie und Pneumologie sind essenziell für eine erfolgreiche Patientenversorgung.

## References

[1] Pichler W, Hausmann O (2016) Classification of Drug Hypersensitivity into Allergic, p‑i, and Pseudo-Allergic Forms. International Archives of Allergy and Immunology 171(3–4):166–179. 10.1159/000453265 27960170

[2] Stirton H, Shear N, Dodiuk-Gad R (2022) Drug Reaction with Eosinophilia and Systemic Symptoms (DReSS)/Drug-Induced Hypersensitivity Syndrome (DiHS)—Readdressing the DReSS. Biomedicines 10(5):999–999. 10.3390/biomedicines10050999 PMC9138571 35625735

[3] Cacoub P, Musette P, Descamps V et al (2011) The DRESS Syndrome: A Literature Review. Am J Med 124(7):588–597. 10.1016/j.amjmed.2011.01.017 21592453

[4] Shiohara T, Mizukawa Y (2019) Drug-Induced Hypersensitivity Syndrome (DiHS)/Drug Reaction with Eosinophilia and Systemic Symptoms (DRESS): An Update in 2019. Allergology International 68(3):301–308. 10.1016/j.alit.2019.03.006 31000444

[5] Schunkert EM, Divito SJ (2021) Updates and Insights in the Diagnosis and Management of DRESS Syndrome. Curr Dermatol Rep 10(4):192–204. 10.1007/s13671-021-00348-z PMC8577395 34777921

[6] Calle AM, Aguirre N, Ardila JC, Cardona Villa R (2023) DRESS Syndrome: A Literature Review and Treatment Algorithm. World Allergy Organization Journal 16(3):100673. 10.1016/j.waojou.2022.100673 PMC10112187 37082745

[7] Spagnolo P, Bonniaud P, Rossi G, Sverzellati N, Cottin V (2022) Drug-Induced Interstitial Lung Disease. European Respiratory Journal 60(4):2102776. 10.1183/13993003.02776-2021 35332071

[8] Hamishegi FS, Singh R, Baruah D et al (2025) Drug-Induced Acute Lung Injury: A Comprehensive Radiologic Review. Journal of Thoracic Imaging. 10.1097/RTI.0000000000000816 39330765

[9] Egli S, Hess T, Goede J (2021) Hypersensitivitäts-Pneumonitis. Swiss Med Forum. 10.4414/smf.2021.08732

[10] Raghu G, Remy-Jardin M, Ryerson C et al (2020) Diagnosis of Hypersensitivity Pneumonitis in Adults: An Official ATS/JRS/ALAT Clinical Practice Guideline. Am J Respir Crit Care Med 202(3):e36–e69. 10.1164/rccm.202005-2032ST PMC7397797 32706311

[11] Brixey AG, Oh A, Alsamarraie A, Chung J (2024) Pictorial Review of Fibrotic Interstitial Lung Disease on High-Resolution CT Scan and Updated Classification. Chest 165(4):908–923. 10.1016/j.chest.2023.11.037 38056824

[12] Wei B, Fox L, Kaffenberger B et al (2024) Drug-Induced Hypersensitivity Syndrome/Drug Reaction with Eosinophilia and Systemic Symptoms. Part II Diagnosis and Management. J Am Acad Dermatol 90(5):911–926. 10.1016/j.jaad.2023.02.073 37516356

[13] Mizukawa Y, Hama N, Miyagawa F et al (2023) Drug-Induced Hypersensitivity Syndrome/Drug Reaction With Eosinophilia and Systemic Symptoms: Predictive Score and Outcomes. J Allergy Clin Immunol Pract 11(10):3169–3178.e7. 10.1016/j.jaip.2023.06.065 37437776

